# A Comprehensive Review and Bifactor Modeling Analysis of the Brief COPE

**DOI:** 10.1177/00469580221108127

**Published:** 2022-10-28

**Authors:** Filipe Rodrigues, Nuno Figueiredo, José Rodrigues, Regina Ferreira, Antonio Hernández-Mendo, Diogo Monteiro

**Affiliations:** 1 ESECS – Polytechnique of Leiria, Leiria, Portugal; 2 Life Quality Research Centre, Rio Maior, Portugal; 3 Sport Science School of Rio Maior – Polytechnique Institute of Santarém (ESDRM-IPSantarém), Rio Maior, Portugal; 4 Health Science School – Polytechnique Institute of Santarém, Santarém, Portugal; 5 University of Málaga, Málaga, Spain; 6 Research Center in Sport, Health and Human Development, Vila Real, Portugal

**Keywords:** cope, mental health, psychometrics, review, bifactor modeling

## Abstract

The Brief COPE is a measure of coping strategies that contains 14 factors. The purpose of this research was twofold: (a) examine the psychometric proprieties of the Brief Cope in previous studies; and (b) perform Confirmatory Factor Analyses (CFA) with second-order model and bifactor model specifications that could be used to assess the best model that represents the 14 coping strategies inherent to the instrument. In order to meet the first objective, a bibliographic review of published peer-reviewed studies between 1997 and 2021 was conducted. Results from the review identified 50 studies, of which 21 used exploratory factor analysis, 28 CFA and one study test-retest analysis. Seventeen studies used the entire correlated 14-factor structure. However, only 11 studies conducted a CFA. For the second objective, a sample of 472 working class individuals (female = 278) with a mean work experience of 19.06 years (SD = 11,92) were recruited. We tested several model specifications, convergent and discriminant validity analysis. We found the correlated 14-factor structure of the Brief COPE to have good psychometric properties. The second-order and bifactor model specifications displayed poor fit or did not converge, respectively. The measure showed good convergent and discriminant validity, and the subscales showed adequate internal consistency. We provide further validity and reliability of the correlated 14-factor structure, evidencing that this measure can assess coping mechanisms. Second-order model specifications need further testing and empirical evidence to support such hierarchical categorization.

**What do we already know about this topic?**
The Brief COPE is a measure of coping strategies that contains 14 factors, and it is the most cited measure of coping in the literature.
**How does your research contribute to the field?**
We provide further evidence on the validity and reliability of the correlated 14-factor structure of the Brief COPE.
**What are your research’s implications toward theory, practice, or policy?**
In practical terms, professionals are recommended to use the Brief COPE with all 14 coping strategies as a way to understand how individuals regulate their behavior during work when faced against stress

## Introduction

The Brief COPE^1^ is a brief form of a previously published instrument called the COPE inventory,^2^ which is a measure of several coping strategies. Specifically, the Brief COPE is a 28-item instrument with 2 items per subscale, and contains 14 subscales measuring active coping, planning, positive reframing, acceptance, humor, religion, using emotional support, using instrumental support, self-distraction, denial, venting, substance use, behavioral disengagement, and self-blame. These factors are based on the model of cope proposed by Lazarus and Folkman^3^ and the Brief COPE measure has been used in diverse contexts and populations. For example, coping has been measured in athletes’ parents,^4^ healthy adults after COVID-19 ease restrictions,^5^ patients with heart failure,^6^ refugees,^7^ or adults exposed to a natural disaster using the Brief COPE.^8^ In fact, this measure was frequently used for multiple types of stressors and participants.^9^

The Brief COPE is the most cited measure of coping in the literature,^9^ compared to other measures of cope such as the Coping Self-Efficacy Scale,^10^ the Brief Resilient Coping Scale,^11^ the Proactive Coping Inventory,^12^ and the Dyadic Coping Inventory.^13^ Kato^9^ cited 765 articles using the Brief COPE and found acceptable reliability, in which the median of the alphas for the measure subscales was .75, with a range from .54 to .91. Hence, it is evident that this measure is popular and has been accepted by the scientific community as a robust measure of cope.

While the Brief COPE is a measure of 14 mechanisms of cope, Carver^1^ has categorized the strategies of acceptance, emotional social support, humor, positive reframing, and religion as emotion-focused coping. The same author proposed active coping, instrumental support, and planning as problem-focused strategies. Finally, behavioral disengagement, denial, self-distraction, self-blaming, and substance use, and venting are dysfunctional coping strategies.^1^ Literature has also considered that coping strategies may be classified as adaptive or maladaptive.^4,14,15^ Depending on several factors, such as context and personality, there is sufficient empirical evidence that points out which are the most related to emotional distress or well-being.^15^ Meyer^14^ classified coping strategies measured by Brief-COPE as maladaptive coping and adaptative coping. According to the mentioned authors, maladaptative coping includes substance use, venting, behavioral disengagement, denial, self-distraction, and self-blame. On the other hand, adaptive coping comprises positive reframing, active coping, acceptance, religion, planning and seeking social support, use of emotional and instrumental support, and humor. This classification has been used in empirical studies.^4,5^ However, results were not consistent. For instance, in the study of Almeida et al^5^ not all adaptive coping strategies were related to well-being.

There is evidence pointing toward a significant correlation between coping mechanisms, comprised within the Brief COPE, and mental health outcomes, as well as well-being indicators. Meyer^14^ found adaptative coping strategies to be negatively related to schizophrenia symptoms and positively related to psychological well-being in patients with severe mental illness. Almeida et al^5^ concluded that adaptive coping was positively correlated with satisfaction with life, while negatively associated with depressive symptoms. On the other hand, maladaptive coping was positively correlated with depressive symptoms, and negatively associated with satisfaction with life. Likewise, García et al^16^ found active coping and acceptance to be positively related with well-being, and negatively related with stress. Kim and Seidlitz^17^ found denial coping (part of maladaptive coping or dysfunctional cope) to be a positive determinant of stress and negative affect. The same authors found problem-focused coping to be positively correlated with positive affect at baseline and 4 weeks later.

There is some of the evidence in the literature showing the positive associations between adaptive coping mechanisms and positive outcomes (eg, well-being, satisfaction with life) and positive correlations between maladaptive coping strategies and negative outcomes (eg, depression, anxiety, stress, negative affect) as pointed out by Kato.^9^ Additionally, there is some evidence that problem-focused coping is positively associated with adaptive outcomes, whereas emotion-focused and mostly dysfunctional coping to be negatively related to adaptive outcomes.^2^

### Review of the Brief COPE

There are several review studies on coping measurements.^9,18,19^ However, there are only 2 review studies in the literature specifically related to the Brief COPE: One of those studies is specifically concerned with the religion factor,^20^ and the other one is a systematic review focused on examining the factor structure of this measure.^21^ Krägeloh^20^ concluded that the results from exploratory factor analyses differ substantially, and that it could be related to the diverse, and often inappropriate, factor analytic techniques used to determine the factor structure of the Brief COPE instrument. Solberg et al^21^ conducted a systematic review of the Brief COPE, aiming to provide validity and reliability of the factor structure. Like Krägeloh^20^ Solberg et al^21^ found that most of the studies were exploratory by nature, using principal component analysis to examine the number of subscales. The referred authors also found that the reports from factor analyses (exploratory and confirmatory) of the Brief COPE are inconsistent with the original model created by Carver.^1^ The mentioned systematic review^21^ showed that only 8 studies provided evidence of the 14-factor model, as other used factor reduction methods. Additionally, Solberg et al^21^ found some critical inconsistencies such as: great variability in the psychometric proprieties; different types of factor analyses (ie, exploratory, confirmatory, and principal component); number of factors; omission or modified items a priori; and the identification of second-order factors is scarce and not always psychometrically valid.

Another limitation of the Brief COPE is the limited information regarding convergent and discriminant validity, which has already been pointed out by Clark et al^22^ related to the COPE measure.^2^ The same limitation is presented in the Brief COPE literature: there is no evidence of convergent and discriminant validity, and several researchers reduced the 14-factor model as a way to obtain acceptable validity.^8,23-25^ Richards et al^26^ pointed out the limitation in their research of lacking convergent and discriminant validity analysis. Additionally, sample size is in some instances too small^27-29^ to analyze correlations between coping strategies and coping outcomes.^9^ Another limitation related to the hierarchical stratification of the coping factors is that there is limited evidence to support it.^14,15^ That is, few studies have conducted factor analysis with second-order model specifications to provide validity of this hierarchical stratification. Without this, existing studies using higher-order models (eg, Teques et al^4^ using the adaptive and maladaptive second-order factors) may be biased by theoretical evidence only. Additionally, none of the existing review studies which are specific to the Brief COPE^20,21^ or which consider this measure (eg, Kato^9^) have reported model fit indices, type of factor analysis, and number of factors of the final model as a means for comparison with the original measurement model.^1^ Besides, analysis of second-order models are scarce.^26,30^

The first objective of this research was to examine the psychometric proprieties of the Brief COPE in previous studies. This objective aimed to explore strengths and limitations of existing studies testing the factor structure of the Brief COPE in different samples. Five databases were searched in order to identify all studies which aimed to validate the Brief COPE to a specific language or population prior to 1997: Google Scholar, Web of Science, PubMED, Academic Search Complete, B-on. The search was conducted using the following terms: Brief COPE AND validation OR scale OR measur* OR instrument. Reference lists of the obtained articles were searched for possible studies.

Inclusion criteria comprised the following: (a) peer-reviewed studies, (b) scale validation studies, and (c) studies using a factor analysis (eg, exploratory factor analysis, confirmatory factor analysis). The database search was open to studies published in English or other languages spanning from 1997 to 2021. The large time span allowed for the inclusion of all articles using the Brief COPE since its development by Carver.^1^

Initial database searching yielded 853 articles, of which 560 were duplicates. Following title and abstract review by the first author, an additional set of 243 articles was removed, resulting in 41 articles meeting inclusion criteria. Cited reference searching yielded 9 additional articles. A total of 50 articles underwent a review and details regarding sample characteristics and psychometric testing results are displayed in Table 1.

**Table 1.** Summary of Sample Characterization and Goodness-of-Fit Indexes of Tested Models Using the Brief COPE.

Authors	Size	Country	Characteristic	Type	Factors	χ^2^/*df*	CFI	TLI	SRMR	RMSEA (CI 90%)
Alghamdi^31^	302	Saudi Arabia	Adults	CFA	3	438.511	.92	.90	NR	.039 (NR)
Amoyal et al^32^	120	USA	Liver transplant candidates	EFA	6	NA				
Amoyal et al^33^	362	USA	Patients with major burn injuries	EFA	7	NA				
Ashktorab et al^34^	212	Iran	Adults	CFA	14	1.06	.97	.99	.04	.02 (NR)
Baumstarck et al^35^	235	France	Cancer Patients	CFA	4	NR	.92	NR	NR	.05 (NR)
Nahlen Bose et al^6^	183	Sweden	Chronic Heart Failure Patients	CFA	4	1.50	.95	NR	.06	.05 (.02, .08)
Brasileiro et al^36^	237	Brazil	Adults	EFA	14	NA				
Carver^1^	126	USA	Adults	EFA	14	NA				
Chase et al^7^	193	Bhutan	Refugees	EFA	5	NA				
Cramer et al^37^	576	USA	Adults	CFA	4	7.72	.86	NR	.08	.11 (.10, .12)
David and Knight^38^	383	USA	Adults	CFA	2	0.70	.97	.92	NR	.06 (NR)
Dias et al^39^	550	Portugal	Adults	CFA	14	1.86	.95	.93	.04	.04 (NR)
Dickstein et al^40^	450	Israel	Adults	EFA	7	2.39	.94	.88	.03	.06 (NR)
Doron et al^41^	2187	France	Adults	CFA	14	3.60	.97	.96	NR	.04 (.03, .04)
Fillion et al^42^	132	Canada	Breast Cancer Patients	EFA	8	NA				
García et al^16^	1847	Chile	Adults	CFA	14	4.16	.92	.91	NR	.04 (.04, .05)
Gutiérrez et al^30^	471	Spain	Patients	CFA	3*	NR	.81	.79	NR	.10 (NR)
Hagan et al^43^	350	USA	Cancer Patients	EFA	7	NA				
Kannis-Dymand et al^8^	674	Australia	Adults Exposed to a Disaster	CFA	4	2.56	.92	.89	NR	.07 (.06, .07)
Kapsou et al^23^	1127	Greek	Adults	CFA	8	3.08	.94	NR	NR	.043 (NR)
Kim and Seidlitz^17^	135	Korea	College Students	EFA	5	NA				
Kimemia et al^44^	134	Kenya	Caregivers	EFA	5	NA				
Knoll et al^24^	110	Germany	Hospitalized Adults and Elderly	CFA	4	1.35	.93	NR	NR	.06
Marôco et al^45^	1573	Portugal	College Students	CFA	14	3.20	.97	.96	NR	.03 (.03, .04)
Mate et al^46^	1204	Spain	Teenagers	EFA	14	NA				
Matsumoto et al^47^	1164	Vietnam	HIV Patients	CFA	6	NR	.87	.85	NR	.07 (NR)
Mejorada et al^48^	203	Mexico	Cancer Patients	EFA	7	NA				
Mohanraj et al^49^	299	India	HIV Patients	CFA	14	NR	.58	NR	NR	.09
Monzani et al^50^	606	Italy	Adults	CFA	14	1.77	.97	.95	.039	.04 (.03, .04)
Muller and Spitz^51^	934	France	College Students	CFA	14	2.34	NR	NR	.03	.04
Muniandy et al^52^	460	Australia	Autistic and Non-autistic adults	EFA	6	NA				
Nawel and Elisabeth^27^	34	Tunisia	Adults	EFA	14	NA				
Nunes et al^28^	153	Portugal	Adults	CFA	14	1.06	.99	.98	NR	.02 (.00, .04)
Perczek et al^53^	148	Spain	Undergraduate Students	EFA	12	NA				
Peters et al^25^	189	America	Pregnant Women	CFA	13	1.77	.93	NR	NR	.06 (.05, .07)
Power et al^54^	377	Canada	Incarcerated adults	CFA	8	7.03	.96	.96	.06	.05 (NR)
Pozzi et al^55^	148	Italy	Patients with Anxiety	CFA	9	NA				
Rahman et al^56^	423	UAE	Adults	CFA	14	3.63	.80	.85	NR	.079 (NR)
Rand et al^57^	1127	USA	Cancer Survivors	CFA	14	3.04	.99	.98	.012	.04 (.04, .05)
Reich et al^58^	203	Uruguay	Adults	EFA	12	NA				
Ribeiro and Rodrigues^59^	364	Portugal	Undergraduate Students	EFA	14	NA				
Richards et al^26^	504	Argentina	Elderly	CFA	2*	3.11	.94	.91	NR	.09 (.07, .12)
Snell et al^60^	147	New Zealand	Traumatic Brain Injury	CFA	9	1.52	NR	NR	NR	.06 (NR)
Su et al^15^	258	China	HIV Patients	EFA	6	NA				
Tang et al^61^	204	China	Adults	CFA	11	1.46	.95	NR	.06	.05 (.04, .06)
Wang et al^62^	190	China	Survivors of Traumatic Events	CFA						
Welbourne et al^63^	190	USA	Adults	EFA	3	NA				
Yuan et al^64^	176	China	Adolescents	EFA	8	NA				
Yuan et al^64^	170	China	Adolescents	CFA	10	1.41	.91	.90	NR	.05 (NR)
Yusoff^65^	90	Malaysia	Adolescents	EFA	9	NA				
Yusoff et al^29^	37	Malaysia	Cancer Patients	Test-retest	14	NA				

*Note*. χ^2^/df = chi-squared test degrees of freedom ratio; CFA = confirmatory factor analysis; EFA = exploratory factor analysis; CFI = Comparative Fit Index; TLI = Tucker Lewis Index; SRMR = standardized root mean square residual; RMSEA = root mean square error of approximation; CI 90% = confidence interval at 90%; NR = not reported; NA = not applicable.

* Second-order factors model using correlated 14-factor model.

Among the 50 reviewed articles, 28 performed a CFA, with 11 having used the 14-factor model. The other studies which included a CFA widely differed from 2 to 13-factor models. Several studies (21) consisted of an EFA, with 5 of them considering the original 14-factor model, and the rest of them ranging from 2 to 12 factors. The remaining study^29^ conducted a test-retest on the 14-factor model. Among the 50 studies, as little as 6 presented all the traditional goodness-of-fit (ie, CFI; TLI; RMSEA; SRMR) indices proposed by Hair et al^66^ Of the 11 studies having used the 14-factor model, 2 displayed poor model fit.^49,56^ However, the remaining studies considering the 14-factor model displayed acceptable to good fit.

As far as the sample size is concerned, 27 studies included less than 300 participants, 10 studies included between 300 and 500 participants, and the remaining 13 studies accounted for more than 500 participants. Only 2 studies explored the second-order model^26,30^ in which only the study of Richards et al^26^ displayed acceptable model fit to the data.

As shown in our bibliographic review, while the Brief COPE has been adapted and validated to different samples and languages, differences in factor structure have emerged, with most studies reducing the number of factors. Hence, while the original Brief COPE^1^ represents a reliable and valid scale, and the 14-factor structure has been consistently evidenced in different studies, there remains a need for exploring second-order structure as proposed by Solberg et al^21^ or even the possibility of a bifactor structure that assesses global experience of coping or specific mechanisms of stress adaptation. Solberg et al^21^ encouraged future research as a means to provide reliable evidence that subscales could indicate a hierarchical structure, as it has been empirically tested in the past (eg, Teques et al^4^). In this study, we sought to psychometrically test the Brief COPE and to examine the validity of a second-order model or bifactor model, and the reliability of this measure in terms of convergent and discriminant validity since, to the best of our knowledge, there is no literature that has conducted these statistical tests. If supported psychometrically, we expect the Brief COPE to be highly beneficial to health psychology, since it is rapidly growing and developing, mostly due to the impact of mental health problems in the working class.^67^ This scale should be very helpful for practitioners who wish to optimize well-being and mental health.

The aim of the present study was to examine the psychometric proprieties of the Brief COPE, testing the original 14-factor structure, as well as second-order models and bifactor model specifications. Additionally, we conducted reliability analysis, as well as convergent and discriminant validity.

## Methods

### Participants

The inclusion criteria to participate in this study were: (a) being at least 18 years old; and (b) agreeing to participate voluntarily in this study. Data were collected between September 2020 and March 2021. A sample of 472 Portuguese working class individuals (female = 278) with a mean work experience of 19.06 years (SD = 11.92) met inclusion criteria and agreed to be respondents for the proposed Brief COPE. They represented a variety of working class (ie, medical staff, sport coaches, nurses, personal trainers, managers, lawyers, etc). Participants completed the Brief COPE Portuguese version^45^ due to the sample characteristics. This version contains all the original 28-items distributed on 14 factors, following the same principle of Likert-type response options anchored between 1 (*Never did this*) and 5 (*Always do this*).

The A-priori sample size calculator^68^ was used for sample size analysis, to calculate the minimum required participants for this study. The following inputs were used: anticipated effect size = .03; desired statistical power = .95; number of latent variables = 14; number of observed variables = 28; probability level = .05. The results suggested a minimum of approximately 292 participants, which provided support that current sample size is acceptable.

### Procedures

Data collection was conducted in accordance with the Declaration of Helsinki and the approval of the Institutional Research Ethics Committee (reference number: CE/IPLEIRIA/17/2021) was obtained prior to data collection. A non-probabilistic sampling technique to collect data was used; specifically, data were collected from a convenience sample of the population. The participants had online access to the questionnaire created for the study using Google Forms, which was promoted using digital media (eg, social networks, academic e-platforms). All participants were informed about the main objective and goals of the study, and 18 years old or older individuals provided written informed consent before completing the questionnaire.

### Statistical Analysis

Analyses were performed in Mplus 7.4.^69^ We considered the Robust Maximum Likelihood estimator to correct any non-normality bias. As previous theoretical^1^ and empirical^16,28,39,45,50,57^ studies gave support for a 14-factor measurement model, we tested the correlated 6-factor model using Confirmatory Factor Analysis (CFA). Specifically, we tested 3 configurations of the factor structure (correlated 2-, 3-, and 14-factor model), 2 second-order factor structure (correlated 2 and 3 higher order-factor model), and 3 bifactor model specifications, (1, 2, and 3 global factors with 14 specific factors). In the CFA and second-order models, items were allowed to load on their predefined factors, suppressing cross-loadings on unintended factors. In bifactor CFA models, items were loaded onto their predefined specific factors and onto specified global factors, and all specific factors were allowed to correlate freely. No missing data at the item level was found due to how the questionnaire was built.

Due to the over-sensitivity of the chi-square statistics on large samples and the model complexity,^66^ we considered several common goodness-of-fit indices to assess model fit, namely: Tucker-Lewis Index (TLI), Comparative Fit Index (CFI), Root Mean Square Error of Approximation (RMSEA) and its respective Confidence Interval at 90% (CI 90%), and Standardized Root Mean Residual (SRMR). For CFI and TLI, values ≥.90 are typically interpreted to reflect adequate fit and for SRMR and RMSEA, values of ≤.080 are indicative of adequate fit to the data.^66,70^

Analysis of the individual items should display significant loadings on the target factor, with weights greater than .50 and significant (*P* < .05), and they should explain at least 25% of the variance.^66^ For the assessment of internal consistency, composite reliability coefficients were calculated for the subscale scores, and values ≥.70 were considered as acceptable.^71^

The Average Variance Extracted (AVE) and the comparison between squared root of the AVE and squared correlations were used to investigate convergent and discriminant validity, respectively. AVE is an established approach to test convergent validity^66^ and scores above .50 are deemed to be acceptable. Constructs are identified as distinct when the square root of the AVE value is larger than the correlation between the 2 constructs displaying discriminant validity.^66^

## Results

Fit indices of the 9 models for the Brief COPE psychometric proprieties are shown in Table 2. The unifactorial, correlated 2- and 3-factor model did not show an acceptable level of fit to the data (CFI and TLI > .90; and SRMR and RMSEA < .080). The correlated 14-factor CFA model displayed adequate fit as seen in Table 2. The 2 and 3 second order models also did not achieve acceptable fit, since CFI and TLI were below cutoff .90, although RMSEA was below .080. All bifactor model specifications did not converge, as the number of iterations exceeded. Thus, we moved on examining factor loadings using the correlated 14-factor model.

**Table 2.** Summary of Goodness-of-Fit indexes for the Tested Models.

Model	Type	*χ* ^2^	*df*	CFI	TLI	SRMR	RMSEA	CI 90%
1. Unifactorial	EFA	4414.510	350	.188	.149	.145	.157	.153, .161
2. Correlated 2 factor^†^	CFA	3835.537*	349	.324	.268	.136	.145	.141, .150
3. Correlated 3 factor^ϕ^	CFA	3587.987*	316	.235	.199	.140	.149	.147, .156
4. Correlated 14 factor	CFA	355.763*	259	.981	.973	.040	.028	.020, .035
5. Correlated 2 second order factors^†^	CFA	924.25*	335	.886	.871	.109	.061	.056, .066
6. Correlated 3 second order factors^ϕ^	CFA	1020.390*	333	.867	.849	.112	.066	.062, .071
7. Bifactor, 1 global factor, 14 specific factors	CFA	DNC						
8. Bifactor, Correlated 2 global factors^†^, 14 specific factors	CFA	DNC						
9. Bifactor, Correlated 3 global factors,^ϕ^ 14 specific factors	CFA	DNC						

*Note*. CFA = confirmatory factor analysis; EFA = exploratory factor analysis; χ^2^ = chi-squared test; *df* = degrees of freedom; CFI = Comparative Fit Index; TLI = Tucker Lewis Index; SRMR = standardized root mean square residual; RMSEA = root mean square error of approximation; CI 90% = confidence interval at 90%; DNC = did not converge.

† Adaptive coping and maladaptive coping factors.

ϕ Problem-focused, emotional-focused, and dysfunctional factors.

* *P* < .001.

Analyses on the correlated 14-factor model (see Table 3) revealed that all item loadings on the target factor were greater than .50 and loaded significantly at *P* < .01. Additionally, responses to each coping mechanism were found to be internally consistent as all factors within the correlated 14-factor model had composite reliability coefficient scores equal or above .70. Specifically, composite coefficients ranged between .70 (planning) and .89 (substance use).

**Table 3.** Factor Loadings, Standardized Errors, and Composite Reliability Coefficients of the Correlated 14-factor Model.

Factors	λ	SE
Active Coping	*0.81*	
Item 1	0.81	0.02
Item 2	0.84	0.03
Planning	*0.70*	
Item 3	0.83	0.03
Item 4	0.61	0.05
Instrumental Support	*0.81*	
Item 5	0.91	0.03
Item 6	0.73	0.04
Emotional Support	*0.86*	
Item 7	0.85	0.03
Item 8	0.88	0.03
Religion	*0.87*	
Item 9	0.94	0.03
Item 10	0.81	0.03
Reframing	*0.85*	
Item 11	0.88	0.03
Item 12	0.84	0.02
Self-blame	*0.73*	
Item 13	0.76	0.04
Item 14	0.75	0.04
Acceptance	*0.74*	
Item 15	0.74	0.07
Item 16	0.79	0.05
Venting	*0.88*	
Item 17	0.91	0.03
Item 18	0.85	0.05
Denial	*0.81*	
Item 19	0.76	0.05
Item 20	0.89	0.05
Self-distraction	*0.82*	
Item 21	0.84	0.04
Item 22	0.82	0.04
Behavioral disengagement	*0.88*	
Item 23	0.89	0.04
Item 24	0.89	0.04
Substance Use	*0.89*	
Item 25	0.90	0.06
Item 26	0.89	0.07
Humor	*0.84*	
Item 27	0.83	0.05
Item 28	0.87	0.04

*Note.* Composite reliability coefficients are in italic.

λ = standardized factor loadings; SE = standard errors.

* *P* < .01.

Convergent validity was achieved as the AVE scores were above .50 as seen in Table 4. According to the squared correlations and square root of AVE scores in Table 4, all factors demonstrated adequate discriminant validity. In general, the correlations of the correlated 14-factor model showed significant associations, specifically: (a) adaptive coping mechanisms were positively correlated with each other and negatively correlated with maladaptive coping strategies and (b) problem-focused coping strategies were positively correlated with each other and negatively correlated with emotional-focused coping strategies and with dysfunctional coping. Details are displayed in Table 4.

**Table 4.** Convergent and Discriminant Validity Analysis.

Factors	AVE	√AVE	1	2	3	4	5	6	7	8	9	10	11	12	13	14
1. Active Coping	.68	.82	1	.59	.12	.05	.04	.36	.00	.01	.03	.02	.00	.12	.00	.05
2. Planning	.53	.73	.77*	1	.17	.08	.03	.31	.00	.18	.08	.01	.01	.08	.00	.03
3. Instrumental Support	.68	.82	.35*	.41*	1	.37	.09	.04	.03	.06	.14	.05	.04	.00	.00	.01
4. Emotional Support	.74	.86	.23*	.29*	.61*	1	.12	.04	.03	.06	.24	.06	.12	.00	.01	.00
5. Religion	.76	.87	.20*	.18*	.30*	.35*	1	.08	.00	.02	.01	.04	.01	.00	.00	.00
6. Reframing	.74	.86	.60*	.56*	.19*	.21*	.29*	1	.01	.23	.01	.00	.03	.04	.00	.18
7. Self–Blame	.57	.75	.01	.03	.16*	.16*	–.07	–.11	1	.01	.23	.01	.03	.04	.00	.18
8. Acceptance	.58	.76	.10*	.42*	.24*	.25*	.14*	.48*	.08	1	.06	.00	.06	.00	.01	.16
9. Venting	.77	.88	.17*	.29*	.38*	.49*	.10	.08	.19*	.24*	1	.09	.07	.00	.04	.02
10. Denial	.68	.82	–.14*	–.10	.23*	.24*	.21*	–.04	.26*	–.02	.30*	1	.09	.08	.07	.01
11. Self–Distraction	.69	.83	.04	.08	.19*	.34*	.10	.16*	.19*	.25*	.27*	.30*	1	.07	.02	.013
12. Behavioral Disengagement	.79	.89	–.35*	–.29*	–.03	.07	.04	–.19*	.22*	–.04	.01	.29*	.26*	1	.12	.02
13. Substance Use	.80	.89	–.06	–.06	.07	.11*	.00	–.02	.26*	.08	.19*	.27*	.13*	.34*	1	.03
14. Humor	.72	.85	.23*	.18*	.12*	.07	.02	.43*	.04	.40*	.13*	.11*	.36*	.14*	.17*	1

*Note*. AVE = average variance extracted; below diagonal line = correlations; above diagonal line = squared correlations.

* *P* > .05.

## Discussion

The present study objective was to analyze the psychometric properties of the Brief COPE (factorial validity, reliability, and construct validity) with a sample of healthy adults. Specifically, the authors examined the dimensionality of the coping strategies based on the original 28-item factor structure, and on doing so, tested a measure of how individuals cope. Current results provide crucial evidence: (a) the 14-factor structure of the Brief COPE was tenable for adults; (b) the proposed factor structure provided acceptable scores of convergent and discriminant validity, as well as reliability; (c) the second-order factor structure did not fit the data; and (d) the bifactor model specifications were not adequate for the data.

The data from the healthy adults fit the 28-item, correlated 14-factor measurement model of the Brief COPE. These results support the psychometrically-sound original scale,^1^ as well as translated versions using the same factor structure.^16,28,34,39,41,45,50,57^ Current results also support specifically those from Marôco et al^45^ and Nunes et al^28^ due to sample characteristics (ie, Portuguese population). Thus, there is evidence that the factor structure of the Brief COPE is suitable and valid for measuring 14 coping strategies, and that measures using alternative factor structure (eg, 3-8 factors) should be revised to explore existing limitations. Specifically, considering that Carver^1^ proposed a valid and reliable measure of 14-factors, practitioners and researchers should adapt the measure according to sample characteristics and not based on statistical tests.

Current results showed that all items loaded significantly onto their predefined factor. In addition, all item loadings on the target factor were greater than .50, explaining at least 25% of variance, which is a good indicator of validity.^66^ Interestingly, several authors^23,25^ removed items or factors for model acceptance. These authors from the several Brief COPE versions claimed low factor loadings, non-significant loading, and cross-loadings as reasons for their removal. These issues could be related to the item meaning (eg, sample characteristics—for instance, atheists could have some difficulties responding to the religion factor), or even sample size (eg, low sample size decreases variability). Another potential reason might be related to the statistical analyses such as using EFA in a previously validated measure for the same language or sample. Nonetheless, Solberg et al^21^ proposed further examination of the Brief COPE, which was achieved in this study.

All our proposed second-order factor models did not provide acceptable fit as seen in our results (Table 2). While the hierarchical factor model with 2 second-order model provided almost acceptable fit (model 5) better than the 3-second order model (model 6), cutoffs were not fulfilled (CFI and TLI > .90; SRMR and RMSEA < .08). Our 2 and 3 second-order models were based on the adaptive and maladaptative coping, and the tripartite aggregation proposed by Carver,^1^ respectively. These results do not support those found by Gutiérrez et al^30^ in which the authors categorized second-order factors as engagement, disengagement, and help-seeking. According to these authors engagement embraces most problem- and emotion-focused strategies, disengagement is close to Carver’s^1^ dysfunctional or less useful coping strategies, and help-seeking coping is characterized by a turn of attention, cognitive processing, and actions toward the social environment. Thus, Gutiérrez et al^30^ created new aggregated based on statistical tests and aggregated inspired by little theoretical and empirical evidence. Richards et al^26^ examining a second-order structure with both coping aggregation, calculated means for each cope factor and targeted to these 2 second-order factors. These authors removed several factors and created a 2-factor model based on model fit: Cognitive active coping (positive reframing, active coping, planning, optimism), different from Gutiérrez et al^30^ and emotional avoidant coping (emotional support, instrumental support, venting, self-distraction). Thus, our results also differ from those reported by Richards et al^26^ since we followed the 2 hierarchical structures most cited and proposed in the literature.^2,4,14,15^ When looking at our results and those provided in the literature in empirical studies,^4,15^ we believe that the 2 second-order factor structure could be acceptable, in some instances, depending on sample characteristics. Our sample was composed by a working-class population, different from parents during sport events^4^ and people living with HIV in China^15^ in which the model fitted the adaptive and maladaptative coping model. Thus, there is evidence that this 2 second-order factor model may suit for some populations.

One of the advantages of the bifactor model specification is that it helps to identify a general factor in addition to the domain specific factors.^72^ Existing literature has never tested such model specification using the Brief COPE, and thus, our study is the first to examine such factor analysis. Regarding the bifactor model specifications, our results showed that the models did not converge. Hence, the bifactor model was not successful in testing the possibility of a unique and broad factor of coping. In result, since all bifactor models did not converge, we are unable to provide information regarding criterion validity. We suggest future research to explore bifactor models with other samples, with other characteristics, as current results could be influenced by our sample.

### Limitations and Agenda for Future Research

Some limitations of the current study should be acknowledged when interpreting the results. This was the first study to examine the psychometric proprieties of the Brief COPE using several second-order and bifactor model specifications, away from the traditional use of EFA. Thus, more empirical studies should be conducted in order to support its results, as well as to replicate the current measure in other cultures, to assess its generalizability. While the sample was relatively large, the labor activities of each individual could have influenced the current results. For example, medical doctors may cope differently compared to coaches and lawyers, and thus, forthcoming studies should explore this possible moderator. In addition, future research should explore variability across contexts and should ascertain whether there is gender invariance in the Brief COPE. Nonetheless, the study employed all crucial methods for scale validity and reliability such as: model fit; factor analysis; correlational analysis between factors; internal consistency analysis; convergent and discriminant validity analysis.

Finally, even though results displayed robust validity and reliability, the associations between cope and adaptive outcomes and negative consequences are still under-researched. For instance, Almeida et al^5^ have shown individuals with adaptive cope to positively predict satisfaction with life, having influence on the depressive symptoms. Hence, future studies are paramount to examine the associations among cope and well-being. So, it is important that a program of research examines the coping strategies to inform policy and practice in the labor context, since the workers’ mental health is crucial for productivity and personal well-being.^73^

## Conclusion

The current study supported the original 14-factor structure of the Brief COPE and its application in the labor context in a sample of healthy working adults. The coping mechanisms were identified again as distinct constructs representing the model of cope proposed by Carver et al^2^ and Carver.^1^ Additionally, this instrument presented itself as a reliable 28-item measure on assessing how adults regulate their behavior during work and how they cope against adverse moments. The reported results provide support for the use of the Brief COPE, adding evidence of the factor structure of this instrument from previous studies.

Our results do not support a second-order model of adaptive and maladaptive cope, neither problem-focused, emotion-focused, and dysfunctional coping, which has been used in previous literature. Differences could be related to sample characteristics, sample size, context, or other artifact variable not controlled during data collection. The bifactor model specification is also open for discussion in future studies, since current results showed statistical problems (ie, model did not converge).

Presented evidence will promote more research examining the stability and validity of the Brief COPE correlated 14-factor structure across a range of context settings and in various cultures. Future research of this scale will help future practitioners and researchers to examine the multidimensionality of coping across populations during labor or other settings such as adverse moments in life (eg, natural disaster, diagnosis of chronic illness). In practical terms, professionals are recommended to use the current measure with all 14 coping strategies to understand how individuals regulate their behavior during work when faced against stress. Hence, practitioners will be able to adapt their behaviors to a more supportive manner, helping individuals to achieve greater well-being and other positive outcomes. Finally, measuring individuals’ coping mechanisms in advance could provide health professionals the required tools to assist on creating supportive environments which could lead to well-being on the long-term.
